# HLA-G+3027 polymorphism is associated with tumor relapse in pediatric Hodgkin's lymphoma

**DOI:** 10.18632/oncotarget.22515

**Published:** 2017-11-18

**Authors:** Valli De Re, Laura Caggiari, Lara Mussolin, Emanuele Stefano d'Amore, Barbara Famengo, Mariangela De Zorzi, Lia Martina, Caterina Elia, Marta Pillon, Nicola Santoro, Paola Muggeo, Salvatore Buffardi, Maurizio Bianchi, Alessandra Sala, Piero Farruggia, Luciana Vinti, Edgardo D. Carosella, Roberta Burnelli, Maurizio Mascarin

**Affiliations:** ^1^ Immunopathology and Cancer Biomarkers, Department of Translational Research, CRO Aviano National Cancer Institute, Aviano, Italy; ^2^ Clinic of Pediatric Hemato-Oncology, Department of Women's and Children's Health, University of Padova, Institute of Paediatric Research Fondazione Città della Speranza, Padova, Italy; ^3^ Department of Pathology, San Bortolo Hospital, Vicenza, Italy; ^4^ Pediatric Radiotherapy Unit, CRO Aviano National Cancer Institute, Aviano, Italy; ^5^ Clinic of Paediatric Hemato-Oncology, Department of Women's and Children's Health, University of Padova, Padova, Italy; ^6^ Department of Paediatric Hemato-Oncology, University of Bari, Bari, Italy; ^7^ Department of Paediatric Hemato-Oncology, Santobono-Pausilipon Children's Hospital, Napoli, Italy; ^8^ Department of Paediatric Hemato-Oncology, Regina Margherita Children's Hospital, Torino, Italy; ^9^ Department of Paediatrics, Ospedale San Gerardo, University of Milano-Bicocca, Fondazione MBBM, Monza, Italy; ^10^ Pediatric Hematology and Oncology Unit, Oncology Department, A.R.N.A.S. Ospedali Civico, Di Cristina e Benfratelli, Palermo, Italy; ^11^ Department of Paediatric Hemato-Oncology, IRCCS Ospedale Bambino Gesù, Roma, Italy; ^12^ Department of Hemato-Immunological Research, Institut des Maladies Emergentes et des Thérapies Innovantes (iMETI), Hôpital Saint Louis, Paris, France; ^13^ Pediatric Hematology-Oncology, Azienda Ospedaliera Universitaria, Ospedale Sant'Anna, Ferrara, Italy

**Keywords:** pediatric hodgkin lymphoma, HLA-G, event-free survival, 3’UTR polymorphism, +3027 C/A genotype

## Abstract

In this study, we tested whether polymorphisms in human leukocyte antigen G (HLA-G) were associated with event-free survival (EFS) in pediatric Hodgkin's lymphoma (HL). We evaluated the association of HLA-G 3′-UTR polymorphisms with EFS in 113 pediatric HL patients treated using the AIEOP LH-2004 protocol. Patients with the +3027-C/A genotype (rs17179101, UTR-7 haplotype) showed lower EFS than those with the +3027-C/C genotype (HR= 3.23, 95%CI: 0.99-10.54, P=0.012). Female patients and systemic B symptomatic patients with the HLA-G +3027 polymorphism showed lower EFS. Multivariate analysis showed that the +3027-A polymorphism (HR 3.17, 95%CI 1.16-8.66, P=0.025) was an independent prognostic factor. Immunohistochemical analysis showed that HL cells from patients with the +3027-C/A genotype did not express HLA-G. Moreover, HLA-G +3027 polymorphism improved EFS prediction when added to the algorithm for therapeutic group classification of pediatric HL patients. Our findings suggest HLA-G +3027 polymorphism is a prognostic marker in pediatric HL patients undergoing treatment according to LH-2004 protocol.

## INTRODUCTION

Hodgkin lymphoma (HL) originates mostly from transformed B-lymphocytes and represents 6% of the childhood tumors [1]. Mononuclear Hodgkin (H) and multinucleated Reed-Sternberg (HRS) tumor cells represent 0.1%–2% of the total tumor infiltrating cells [2]. A substantial number of HL cases are linked to Epstein-Barr virus (EBV) [3]. A follow-up clinical study of the Italian Association of Pediatric Hematology-Oncology (AIEOP) trial LH-2004 for pediatric HL showed that 108 out of 769 (14%) enrolled children showed disease progression or relapse (37 progression and 71 relapses), in general 2 years after diagnosis [4]. Therefore, it is important to identify markers that predict relapse in HL patients in order to obtain better responses with less aggressive therapy, particularly considering the long term adverse effects of HL therapy such as secondary cancer, infertility and neurological toxicity [5]. Genetic instability is also associated with HL. Therefore, it is key to identify genetic factors that are involved in HL pathogenesis and response to therapy [6–7].

Class I histocompatibility leukocyte antigen (HLA) molecules play an important role in the ability of tumor cells to evade immune surveillance [8]. Moreover, natural killer cells (NK) that kill cancer cells expressing HLA class I, are locally inhibited in HL [9–10]. Therefore, antigen-presentation by the HLA complex and co-stimulatory molecule expression plays a key role in the etiology of HL, including familial aggregation and the epidemiology of HL [11–15]. However, it is unclear if HLA genes are associated with HL due to high degree of polymorphism, peculiar genomic distribution and high level of linkage disequilibrium (LD) among the HLA genes [16].

HLA-G is a non-classical HLA class I molecule that is expressed in physiologic conditions in immune privileged tissues (e.g. cornea, thymus, pancreatic islets, endothelial cell precursors, and erythroblasts) [17]. The alternate splicing of HLA-G transcript generates seven different mRNAs that give rise to four membrane-bound (HLA-G1, G2, G3 and G4) and three soluble (HLA-G5, G6 and G7) proteins [18–21]. Both membrane-anchored and soluble HLA-G forms generate strong inhibitory signals after interaction with their cognate receptors ILT2, ILT4 and KIR2DL4, which are differentially expressed by NK, T cells, and antigen-presenting cells [22]. HLA-G coding region has limited variability except for the 5′upstream regulatory region (5′UTR) [23] and the 3′ untranslated region (3′ UTR) [24], both of which influence the HLA-G expression. HLA-G plays an important role in maintaining immune tolerance during pregnancy [17]. HLA-G is induced during transplantation [25, 26], viral infection [27] and malignancies [28, 29] and its expression correlates with the size and aggressiveness of solid tumor [30, 31]. HLA-G is also expressed by immune cells in the tumor microenvironment by trogocitosis, thereby restricting their local proliferation and cytotoxicity [32]. Furthermore, its expression is regulated by several factors such as IL-10, IFN-γ, heat shock, hypoxia, oxidative stress and radiation, which further impacts immune response towards tumor and tumor-infiltrating cells [33]. HLA-G expression and its clinical significance has been extensively investigated in solid tumors, but its role in hematological malignancies has not been well established [34]. Many differences have been reported between solid and hematological cancers. These differences are probably because immune cells, unlike epithelial cells, express both HLA-G and HLA-G receptors (ILT2) that inhibit proliferation of B-cell lymphomas, myelomas, and B-cell leukemia [35]. HLA-G expression in classic HL patients has been investigated in 2 studies both regarding adult patients. These authors showed about 50% of positive- tumor cells for HLA-G expression and suggested that induction of HLA-G protein contributed to the modulation of immune responses observed in HL since HLA-G expression correlated with a better prognosis [36, 37]. Thus, in hematological diseases, the role of HLA-G is more complex than in solid tumors and depends on the balance between inhibition of anti-tumor responses and the anti-proliferative effects on malignant B cells [28, 35]. Since it is important to understand the significance of HLA-G expression in hematological malignancies, we investigated the association of HLA-G 3′-UTR polymorphisms with event-free survival (EFS) in pediatric HL patients.

## RESULTS

### Patient and disease characteristics

Patient and disease characteristics are listed in Table 1. The HL patients were sub-divided into classical HL subtypes such as nodular sclerosis (cHL-NS n=86), mixed cellularity (cHL-MC, n=7), nodular lymphocyte-predominant (HL-LPn, n=9) and unspecified (HL, n=11) according to the WHO guidelines [38]. The median follow-up period was 3.5 y. Based on Ann Arbor staging, patients were subdivided into stages I (n=7), II (n= 54), III (n=22) and IV (n=30). Twenty-seven patients (24%) showed relapse or tumor progression during the follow-up and two patients (1.7%) developed secondary tumor.

**Table 1:** Demographic and clinicopathological characteristics of HL patients

Characteristics	Total N. of patients (n =113) (%)
Age at diagnosis, years	
Median	13.5
Range	3-18
Mean	13.2
Sex	
Female	45 (40)
Male	68 (60)
WHO classification	
HL-LPn	9 (8)
cHL-NS	86 (76)
cHL-MC	7 (6)
HL	11 (10)
Symptoms	
A	54 (48)
B	59 (52)
Secondary tumor	
Yes	2 (2)
No	111 (98)
Ann Arbor Staging	
I	6 (5)
II	54 (48)
III	22 (20)
IV	31 (27)
Therapeutic risk group	
1	17 (15)
2	16 (14)
3	80 (71)
Treatment outcome	
Complete remission	86 (76)
Events (progression or relapse)	27 (24)
Tumor released	
YES	22 (19)
NO	91 (81)
Observation time	
Mean	4.58
Median	3.54
Range	0.19-16.48

HL-LPn=nodular lymphocyte predominant type; cHL-NS=nodular sclerosis; cHL-MC=mixed cellularity; HL= HL not easily classified.

### *HLA-G +3027* polymorphism is associated with EFS after AIEOP LH-2004 treatment

As shown in Supplementary Table 1 the genotype and allele frequencies of eight 3′UTR HLA-G polymorphisms [14-bp ins/del (rs371194629), +3003C/T (rs1707), +3010C/G (rs1710), +3027C/A (rs17179101), +3035C/T (rs17179108), +3142C/G (rs1063320), +3187A/G (rs9380142) and +3196C/G (rs1610696)] were similar in control subjects or blood donors (BD; n=259) and HL patients (n=113). In the control subjects, there were no differences in the allele and gene frequencies based on age or gender for any of the eight polymorphisms (data not shown). However, subgroup analysis of HL patients by (i) gender showed that male gender was associated with the +3187-AA genotype, (ii) the +3010-CC+CG; +3027-AA+CA and +3142-GG+CG genotypes were associated with increased risk for EFS, (iii) the +3196-CC genotype correlated with higher Ann Arbor stages (Table 2).

**Table 2:** Association of HLA-G 3′UTR polymorphisms with gender and clinicopathological characteristics of pediatric HL patients

GENDER	Femalen= 45 (%)	Malen= 68 (%)	OR(95% CI)	*P*	
**+3187A/G**					
A	53 (59)	101 (74)	1.00	***0.016***	
G	37 (41)	35 (26)	0.49 (0.28-0.89)		
AA	15 (33.3)	38 (55.9)	1.00	***0.018***	
AG+GG	30 (66.7)	30 (44.1)	0.39 (0.18-0.86)		
**Treatment outcome (tumor relapse)**	**Event (No)**	**Event (Yes)**	**OR(95% CI)**	***P***	
**+3010C/G**					
C	91 (51)	30 (68)	1.00	***0.027***	
G	89 (49)	14 (32)	0.45 (0.21-0.94)		
CC+CG	69 (76.7)	21 (95.5)	1.00	***0.025***	
GG	21 (23.3)	1 (4.5)	0.16 (0.02-1.23)		
**+3027C/A**					
C	170 (94)	37 (84)	1.00	***0.026***	
A	10 (6)	7 (16)	3.13 (0.10-8.94)		
CC	82 (91.1)	15 (68.2)	1.00	***0.010***	
AA+CA	8 (8.9)	7 (31.8)	4.78 (1.51 −15.17)		
**+3142C/G**					
C	92 (51)	30 (68)	1.00	***0.031***	
G	88 (49)	14 (32)	0.45 (0.21-0.95)		
GG+CG	70 (77.8)	21 (95.5)	1.00	***0.032***	
CC	20 (22.2)	1 (4.5)	0.17 (0.02 −1.32)		
**Ann Arbor Stages**	**Stage I**	**Stage II**	**Stage III**	**Stage IV**	P^*^
**+3196C/G**					
C	8 (66.7)	72 (66.7)	28 (63.6)	52 (83.9)	***0.033***
G	4 (33.3)	36 (33.3)	16 (36.4)	10 (16.1)	
CC	2 (33.3)	24 (44.4)	10 (45.5)	20 (66.7)	***0.021***
CG+GG	4 (66.7)	30 (55.6)	12 (44.5)	10 (33.3)	

OR=odds ratio; 95%CI=95% confidence interval.

P^*^ chi-square test for trend.

CTRL: controls of individuals without hematological malignancies.

We observed that the +3010-C, +3027-A and +3142-G alleles were part of the unique 3′-UTR-7 haplotype of *HLA-G* encompassing the +3027-A variant [24]. Therefore, we chose the +3027 polymorphism rather than UTR-7 haplotype for further analysis.

**Figure 1:**
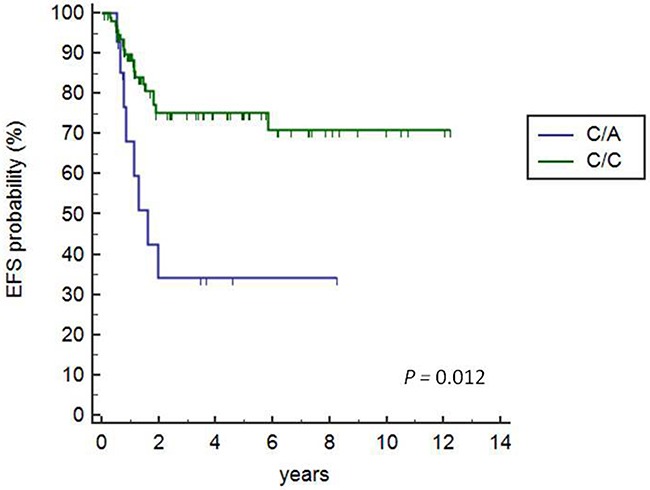
Kaplan-Meier survival analysis of +3027C/A HLA-G polymorphisms Kaplan Meier survival curves show 72.3% and 34% EFS for patients with the +3027 C/C and C/A variant, respectively (C/A vs. C/C, HR=3.23, 95% CI 0.99-10.54; P=0.012). The solitary patient with A/A genotype was excluded from the analysis.

We found that 27 (23.9%) of the 113 patients that received AIEOP LH-2004 treatment experienced a tumor-event in 1.2±1.048 y (range 0.4-6 y) after diagnosis. Kaplan Meier survival analysis demonstrated that the patients carrying the heterozygous +3027-C/A genotype showed lower EFS time than patients carrying the C/C genotype (HR=3.23, 95%CI: 0.99-10.54; P=0.012; Figure 1). Only 1 out of 113 patients carried the A/A genotype and were excluded from the analysis.

### HLA-G +3027 A variant is an adverse independent risk factor for EFS

**Figure 2:**
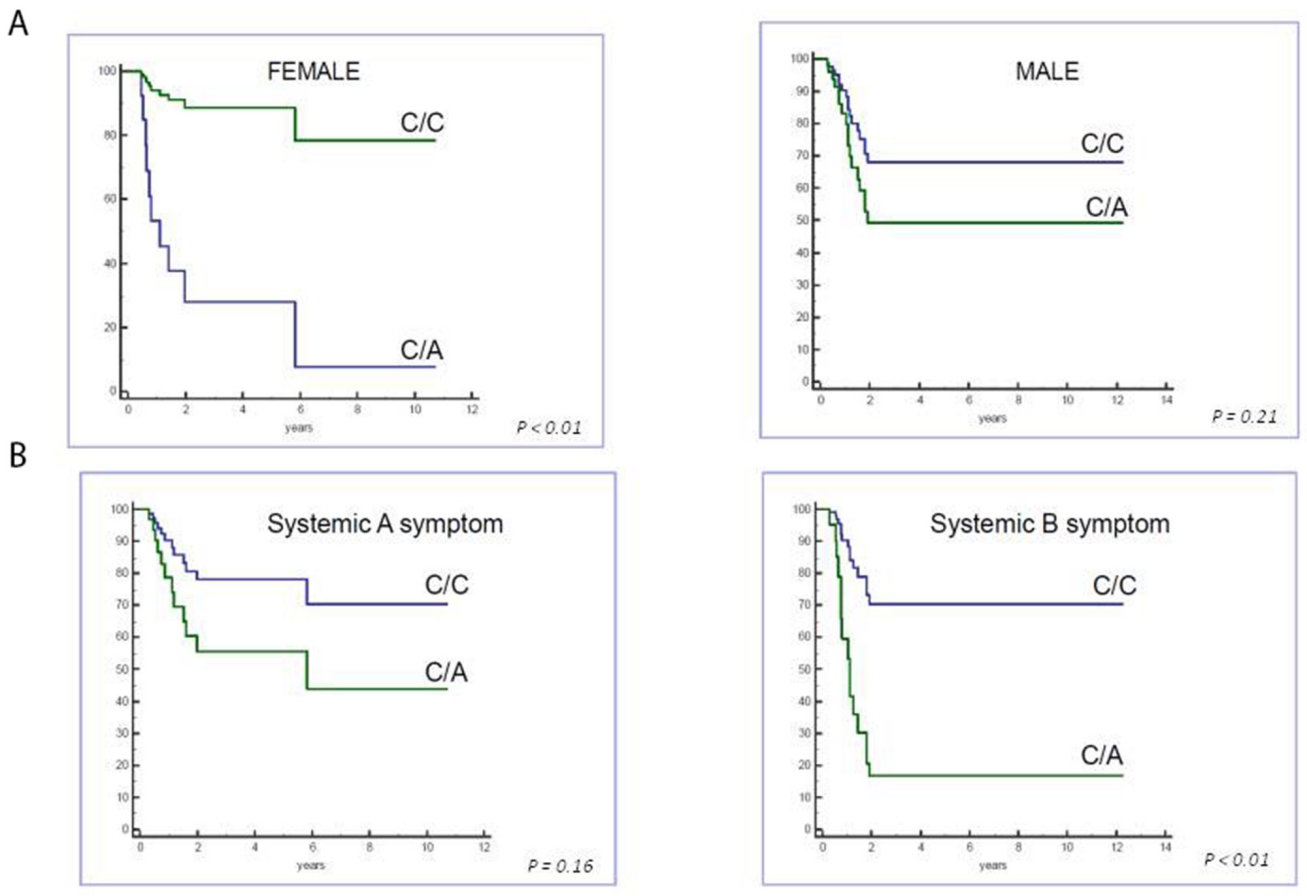
Cox regression analysis of event-free survival based on gender and systemic symptoms Cox regression analysis shows patients carrying +3027 HLA-G polymorphism show lower EFS in **(A)** female and **(B)** systemic B symptomatic patients.

Multivariate analysis by Cox proportional hazard regression for HLA-G polymorphisms (+3010, +3027, +3142, gender, age and therapeutic group classification) demonstrated that +3027-A polymorphism (UTR-7 haplotype) was an independent prognostic factor for poor EFS (P=0.025; HR=3.17, 95% CI: 1.16-8.66; Table 3). Moreover, female gender and systemic B symptoms in combination were associated with poor EFS in pediatric HL patients with the +3027 A (UTR-7) haplotype (Figure 2).

**Table 3:** Univariate and multivariate Cox regression analysis of +3027 HLA-G polymorphism as a risk factor in pediatric HL patients

Variable	Univariate analysis			Multivariate analysis		
	*P-value*	HR	95%CI	*P-value*	HR	95%CI
+3027 HLA-G						
C/C	*--*	1.00	--	*--*	1.00	--
C/A	***0.006***	3.23	1.415 - 7.36	***0.0251***	3.17	1.16-8.66

CI=confidence interval; HR=1.00 for reference category.

The following co-variables were tested: +3010, +3027, +3142 polymorphisms, gender, age, histological WHO classification and AIEOP LH-2004 classification for treatment response.

The +3027 A-variant or the UTR-7 haplotype encompasses the 14bp region that spans the Ins, +3003T, +3010C, +3027A, +3035T, +3142G, +3187A and +3196C polymorphic sites. Only significant results obtained in multivariate analysis were reported.

### Inclusion of HLA-G +3027 polymorphism enhances EFS risk estimation by the clinical therapeutic group (GR)

According to the AIEOP LH-2004 criteria, patients were stratified into 3 therapeutic groups (GR) based on increased radiation dosage and aggressive chemotherapy (both length and intensity) to reduce long-term toxicities in younger patients [4]. The groups according to AIEOP LH-2004 risk score criteria were (1) GR1: stage IA or IIA without mediastinal bulky disease or involvement of lung hilum lymph nodes, and less than four lymph nodal regions; (2) GR2: negative for GR1 or GR3 criteria; (3) GR3: stage IIIB or stage IV or mediastinal bulky disease. Therapy was performed based on interim or post-chemotherapy positron emission tomography (PET) analysis by quantitative Fluorodeoxyglucose (FDG) avidity of tumor mass.

**Figure 3:**
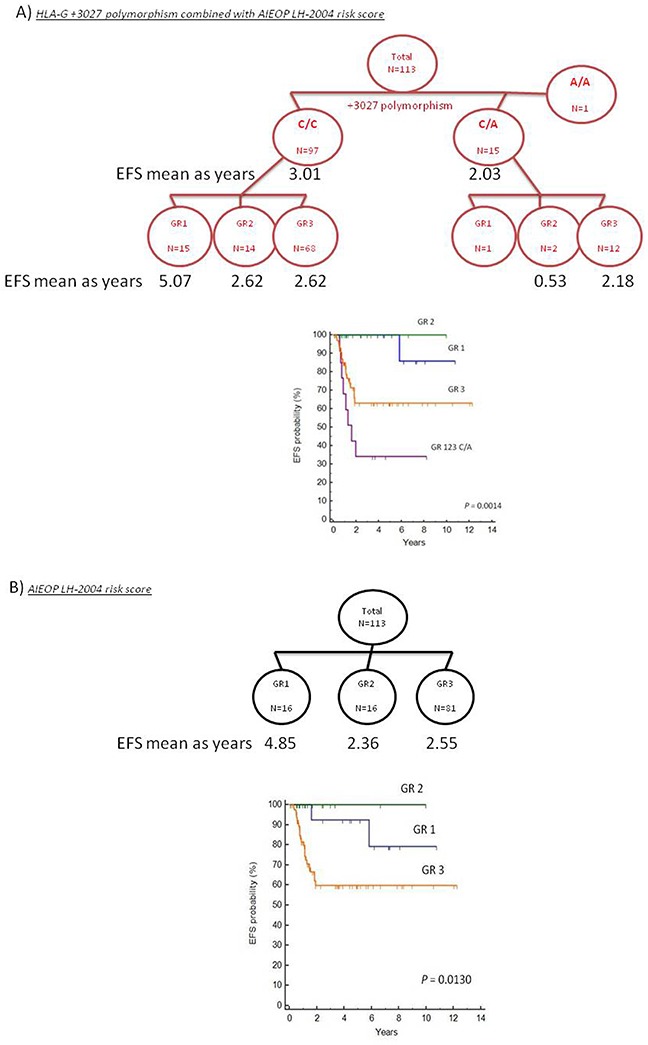
Kaplan Meier survival analysis comparing algorithms based on the HLA-G genotype and AIEOP LH-2004 risk score classification **(A)** Kaplan Meiersurvival curves based on HLA-G +3027 algorithm is shown. Patients were divided into 2 groups based on their HLA-G +3027 genotype (C/C and C/A) and further divided into 3 groups based the AIEOP LH-2004 therapeutic risk score (GR). Since the GR1 and GR2 groups with the +3027 C/A variant included less than 2 patients, GR1, GR2 and GR3 groups with the +3027 C/A variant were merged to form a unique GR123-C/A group. **(B)** Kaplan Meier survival curves based on the AIEOP LH-2004 risk score for therapeutic response (GR) in pediatric HL patients. Note: Criteria for the AIEOP LH2004 therapeutic group (GR) classification are in the methods section.

**Figure 4:**
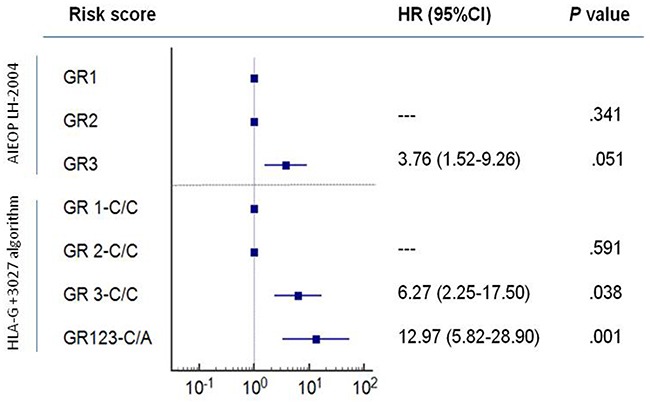
Comparison of the risk values based on AIEOP LH-2004 group therapy protocol and HLA-G +3027 algorithm Kaplan-Meier survival analysis show hazard ratios (HR) for GR1, GR2 and GR3 groups based on AIEOP LH-2004 therapy and the HLA-G genetic groups, GR1,2,3-C/C and GR123-C/A based on our analysis. Note: GR1 and GR 1-C/C as reference categories; 95% CI confidence intervals are shown for event-free survival (EFS).

In the present study, we included the HLA-G +3027 risk factor in the algorithm with the AIEOP LH-2004 GR score to predict the EFS risk (Figure 3A). We performed Kaplan-Meier analysis of this new algorithm and compared it to AIEOP LH-2004 GR score alone (Figure 3B). Since the GR1 and GR2 groups included less than 2 patients with +3027 C/A variant, we merged the GR1, GR2 and GR3 groups with the +3027 C/A variant as a unique GR123-C/A group. We stratified patients in the 4 categories with lower EFS by the presence of +3027-C/A genotype as shown in the Kaplan-Meier analysis (Figure 3). Based on the +3027 genetic algorithm, 8 out of 15 patients (53%) with the +3027-C/A genotype, and 18 out of the 68 patients (26%) in the GR3 category showed lower EFS (≤2years). We compared the hazard ratio (HR) values obtained for both AIEOP LH-2004 GR and HLA-G +3027 algorithms and found better risk stratification with the HLA-G +3027 algorithm (Figure 4).

### Overall survival estimates based on the HLA-G +3027 algorithm

**Figure 5:**
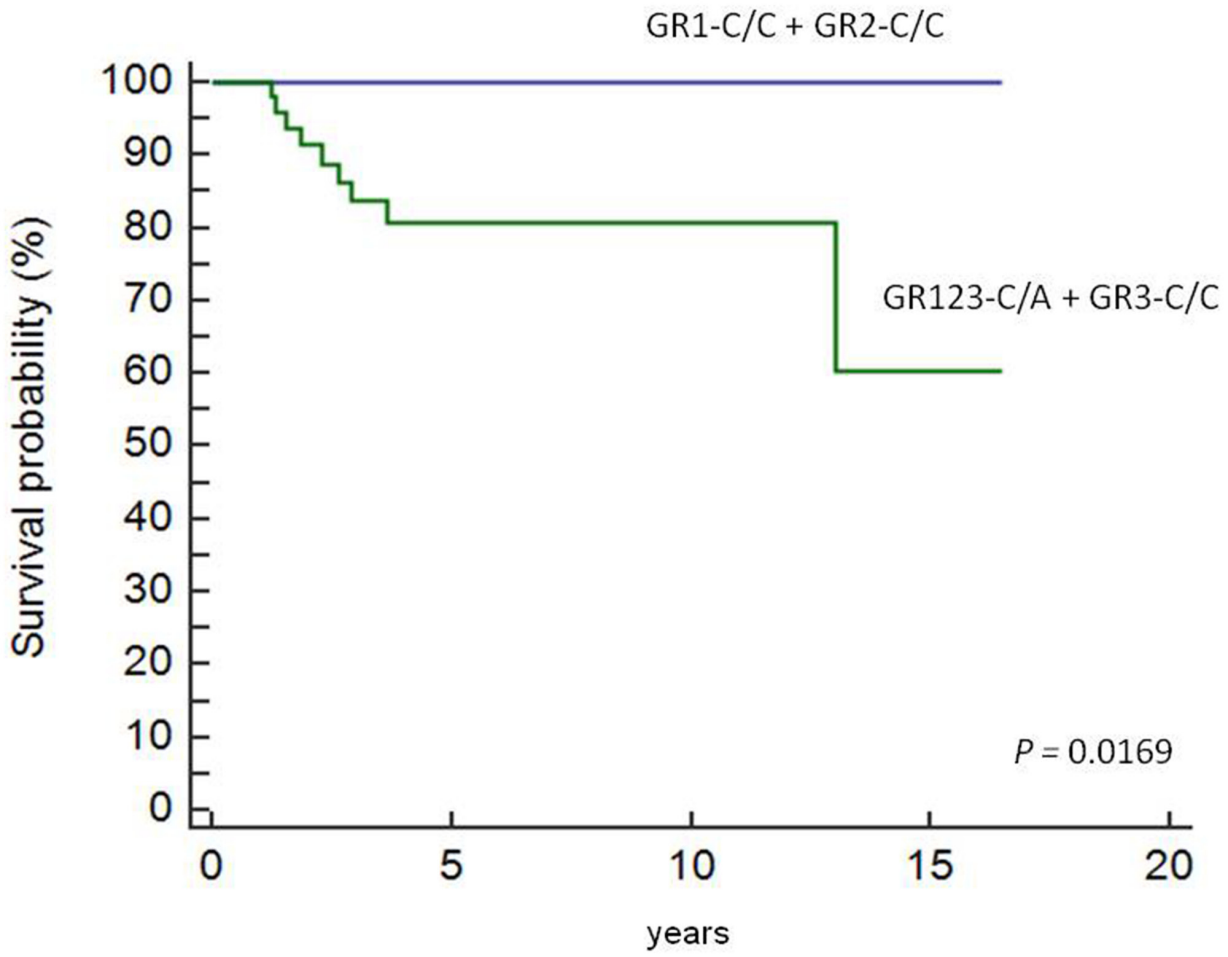
Overall survival in pediatric HL patients based on therapeutic grouping Cox regression analysis showing OS in pediatric HL patients (n=113) based on therapeutic grouping (GR). As shown, GR1-C/C and GR2- C/C patients show higher EFS than GR3-C/C and GR123-C/A patients.

Cox regression analysis showed that patients in the GR123-C/A and GR3-C/C groups were associated with decreased overall survival and lower EFS score according to the HLA-G +3027 algorithm (Figure 5). Nine of the 113 patients that died belonged to the GR123-C/A and GR3-C/C groups.

### Analysis of HLA-G Expression in malignant cells

**Figure 6:**
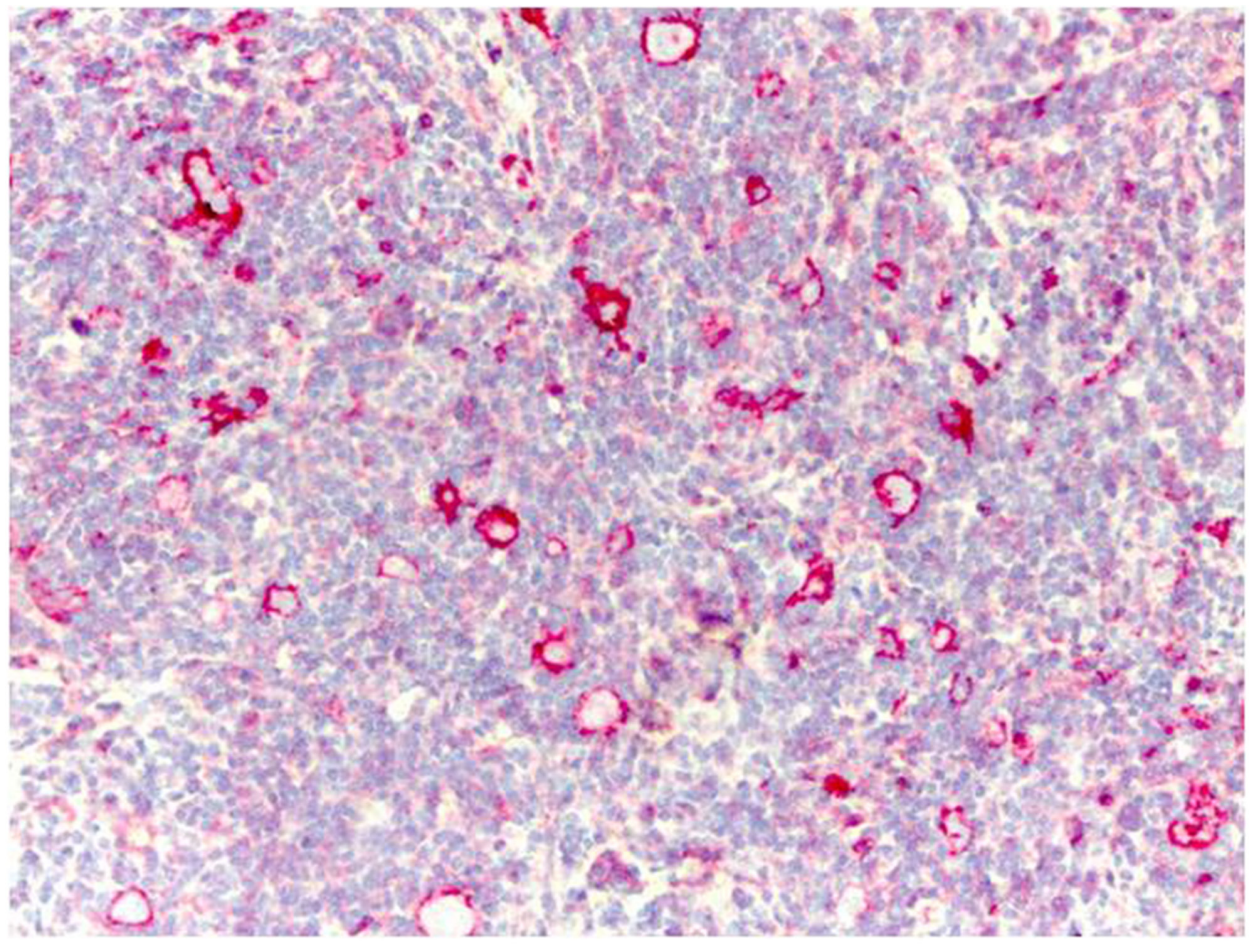
Immunohistochemical analysis of HLA-G protein expression in Reed-Sternberg cells in pediatric HL patients Representative image shows immunohistochemical staining for HLA-G in a HL patient carrying the wild type C/C +3027 genotype. Formalin/PFA-fixed paraffin-embedded sections were stained with primary anti-HLA-G antibody (4H84). Lymphoma cells show strong membrane staining for HLAG. Note: Only 5 out of 25 patients analyzed were positive for HLA-G in our analysis.

Immunohistochemical analysis of HLA-G expression in tumor cells from 25 lymph node biopsies that were obtained at diagnosis showed that patients carrying a +3027 C/A genotype were all negative for HLA-G and only 5 cases having the wild type +3027 C/C genotype showed HLA-G expression (20%; Figure 6).

## DISCUSSION

In this study, we showed that the +3027-A (UTR-7 haplotype) HLA-G polymorphism was associated with a low EFS in pediatric HL patients treated according to the AIEOP LH-2004 protocol, more evident in female gender and patients with systemic B symptoms. Multivariate analysis demonstrated that +3027-C/A genotype was an independent prognostic factor associated with poor EFS. Moreover, incorporating the HLA-G +3027 polymorphism into the AIEOP LH-2004 therapeutic stratification improved risk evaluation. Patients belonging to the GR123-C/A and the GR3 groups showed lower 5 year-overall survival than patients belonging to GR1 and GR2 groups (9.7% vs. 0%). The effect of HLA-G expression on HL patient survival needs further investigation.

Many studies have reported association of polymorphisms in the HLA region with HL [39, 40]. However, the association of variants in the 3′UTR of HLA-G with clinical outcome in HL has not been reported so far. Moreover, mechanisms that regulate HLA-G expression in B-cells are not well defined [37, 41, 42]. We demonstrated that HLA-G was not expressed in the tumor cells of HL patients with +3027-C/A genotype and present only in a minority of cases (20%) with the wild type +3027-C/C genotype. Previously, HLA-G expression was reported in adult HL patients belonging to Ann Arbor stages IIb-IV in the tumor microenvironment in patients showing positron emission tomography (PET)-positive (carried out after 2 cycles of standard chemotherapy) during follow-up [37]. Conversely, PET-negative patients showed higher HLA-G protein expression levels on tumor cells (50% of cases) suggesting that HLA-G protein expression inhibited tumor progression [37]. We demonstrated that HLA-G was not expressed in the lymph node of HL patients carrying the HLA-G +3027 C/A genotype. However, HLA-G was expressed in few cases (n=5) with +3027 C/C genotype and therefore, the role of the wild type HLA-G C/C genotype needs to be studied in greater detail. Caocci *et al.* showed that HLA-G expression was a positive prognostic factor in patients with advanced-stage classical HL [37]. In the present study, we found HLA-G-positive tumor cells in lower number of cases (n=5/25, 20%) than reported by Diesptra *et al*. and Caocci *et al*. (about 55%). However, all of our HLA-G-positive cases demonstrated positive prognosis as reported earlier. Patients that carried the +3027 C/A variant were associated with poor prognosis and were all HLA-G-negative.

HLA-G expression can exert beneficial or detrimental effects of immune recognition, which depend on the pathological context or situation [34, 43]. HLA-G expression suppresses immune response in case of allotransplantation and autoimmune disorders [44]. However, HLA-G expression would be detrimental in solid tumors and virally infected cells, where it suppresses immune response by inhibiting NK cells that kill tumor cells [45]. Correlation between HLA-G expression and clinical outcome in hematological malignancies is unclear [46]. One probable reason is that contrary to epithelial cells, immune cells express both HLA-G and its inhibitory receptors such as ILT-2 on their surface. In multiple myeloma and B-cell leukemia, interaction with HLA-G and ILT2 inhibitory receptor induces tumor cell apoptosis [35]. Moreover, since the HLA-G +3027-A variant was found associated with lower HLA-G expression [41], our data might suggest that patients with the +3027-C/A genotype are more prone to tumor relapse because more prone to decreased expression in HLA-G, which affects HL tumor cell apoptosis.

Of note, the impact of chemotherapy and radiotherapy on HLA-G expression in patients carrying the HLA-G +3027-C/C and the HLA-G +3027-C/A genotypes is not known. Previous reports have shown that gamma radiation and/or chemotherapy modulates surface HLA-G expression in B-cell lines (M8 and K562), melanoma cell lines (OCM-1A and JEG-3) and basal cell carcinoma of the skin [47, 48]. Thus, it is possible that chemo/radiotherapy modulate HLA-G expression also in our patients having a good prognosis, but not in patients having the HLA-G +3027 C/A genotype for a reason still now unknown.

However, it is also possible that +3027-C/A polymorphism could be in strong linkage disequilibrium with another critical gene, which causes the disease progression and poor EFS.

Overall these issues needs to be investigated in detail.

In conclusion, we demonstrated that +3027-A HLA-G polymorphism was associated with poor EFS in pediatric HL patients treated with the AIEOP LH-2004 protocol. In the future, comprehensive prospective studies are necessary to confirm that HLA-G polymorphisms are prognostic indicators in pediatric HL therapy.

## MATERIALS AND METHODS

### Sample collection and Patient grouping criteria

Peripheral blood samples were collected from 113 pediatric HL patients (range 3-18 years) at the AIEOP Centers in the laboratory of Padua University from December 2004 to August 2014. Table 1 lists patient information regarding age, sex, the presence of a secondary tumor, relapse and/or progression tumor events, presence or absence of systemic symptoms and Hodgkin's lymphoma subtype as recommended by World Health Organization (WHO) [38]. The patients were also staged according to Ann Arbor classification [48] and allocated into 3 therapeutic groups (GR) based on risk [4]. These 3 groups were (1) GR1: stage IA or IIA without mediastinal bulky disease or involvement of lung hilum lymph nodes, and with less than four lymph nodal regions; (2) GR2: absence of criteria for GR1 or GR3; and (3) GR3: stage IIIB or stage IV or mediastinal bulky disease. The patients were treated according to the protocol approved for the AIEOP multicenter clinical trial LH-2004 [4].

### Treatment protocol and follow-up

The GR1 patients (low risk) were administered 3 cycles of ABVD (Adriamycin, bleomycin, vinblastine, dacarbazine) without radiotherapy (RT) were administrated. In case of unsatisfactory response after 2 cycles of ABVD or residual disease after 3 cycles, GR1 patients received chemotherapy/RT or RT. The GR2 (intermediate risk) and GR3 (high risk) patients were administered 4 or 6 alternating cycles of COPP-ABV chemotherapy (cylcophosphamide, vincristine, procarbazine, prednisone and ABV) to ABVD treatment. Radiotherapy was administered with Involved Field Technique (IFRT) at cumulative dose of 14.4 or 25.2 Gy depending on the remission status (complete or partial) at the end of chemotherapy. Patients in complete remission at the end of chemotherapy were not administered radiotherapy. Complete response (CR) was defined as absence of clinical, radiological (ultrasound and/or CT scan evaluation) and FDG-PET evidence of disease. We enrolled 259 local blood donors (BD) as a control group. Study was approved by the ethics committee of all participating institutions. Written consent forms were obtained from parents or legal guardians of all patients.

### HLA-G genotyping

Genomic DNA was extracted from peripheral blood with the Qiagen DNAeasy Kit (QIAGEN, Grand Island, NY). HLA-G genotyping was performed by first PCR amplifying using the 3′UTR specific primers (forward, 5′-TGTGAAACAGCTGCCCTGTGT-3′ and reverse, 5′-GTCTTCCATTTATTTTGTCTCT-3′) [24] followed by Direct BigDye Terminator sequencing. The data was analyzed with the Assign SBT software version 3.27b (Conexio Genomics, Fremantle, Western Australia) [49]. All polymorphic sites were individually annotated.

### Immunohistochemistry

Paraffin-embedded primary HL lymph node tissue was retrieved. HLA-G expression was assessed in 25 paraffin-embedded tissues by Immunohistochemical staining with primary 4H84 mouse monoclonal antibody (Abcam, HIER, 1:2000), which recognizes all the HLA-G isoforms. If intense membrane HLA-G staining was found in more than 10% of Hodgkin or Reed-Sternberg cells, then the sample was scored as positive.

### Statistical analysis

The frequencies of HLA allele and genotype were compared between patients and controls chi-square test (VassarStats http://faculty.vassar.edu/lowry/VassarStats.html and the SKDM software (http://sourceforge.net/projects/skdm). The HLA-G 3′UTR haplotypes were defined according to Castelli [50]. HLA haplotype frequencies were estimated by the ARLEQUIN ver 3.1 software [51]. Frequencies and distributions of clinical characteristics were calculated as the period of time after diagnosis. Kaplan-Meier survival analysis was performed to determine relapse rate and overall survival after diagnosis and treatment according to the LH-2004 protocol. We included age, gender, Ann Arbor stage, tumor response, presence of systemic symptom and WHO histopathological classification as potential co-variables in the univariate model. Multivariate Cox proportional hazards models were used to estimate hazard ratios (HRs) for EFS with 95% CIs adjusted for +3027 polymorphism and potential co-variables. The most frequent variable was automatically selected as the reference category. Clinical prognostic risk groups [4] currently used to stratify therapy were used to compare the +3027 HLA-G genetic stratification.

## SUPPLEMENTARY MATERIALS TABLE




