# Supplementary material for: HLA-G+3027 polymorphism is associated with tumor relapse in pediatric Hodgkin's lymphoma
Source: Oncotarget. 2017 Nov 18;8(62):105957–70. doi: 10.18632/oncotarget.22515 (PMC5739693; doi:10.18632/oncotarget.22515)
Supplement: Supplementary file 1 [file oncotarget-08-105957-s001.pdf]

HLA-G+3027 polymorphism is associated with tumor relapse in pediatric Hodgkin's lymphoma

SUPPLEMENTARY MATERIALS

Supplementary Table 1: Genotype and allele frequencies of 8 HLA-G 3' UTR polymorphic variants (14 bp Ins/Del, 3003C/T, 3010C/G, 3027A/C, 3035C/T, 3142C/G, 3187A/G, 3196C/G) in 259 controls (blood donors, BD) compared to 113 patients with Hodgkin lymphoma (HL).

See Supplementary File 1
