# Supplementary material for: HLA-G+3027 polymorphism is associated with tumor relapse in pediatric Hodgkin's lymphoma
Source: Oncotarget. 2017 Nov 18;8(62):105957–70. doi: 10.18632/oncotarget.22515 (PMC5739693; doi:10.18632/oncotarget.22515)
Supplement: Supplementary file 2 [file oncotarget-08-105957-s002.docx]

**Supplementary Table 1: Genotype and allele frequencies of 8 HLA-G 3’ UTR polymorphic variants (14 bp Ins/Del, 3003C/T, 3010C/G, 3027A/C, 3035C/T, 3142C/G, 3187A/G, 3196C/G) in 259 controls (blood donors, BD) compared to 113 patients with Hodgkin lymphoma (HL).**

| **HLA-G 3’ UTR Polymorphism** | **BD**  **n= 259** | ***Frequency*** | **HL**  **n= 113** | ***Frequency*** | ***P-*value** | **OR. 95% CI** |
| --- | --- | --- | --- | --- | --- | --- |
| **14-bp** |  |  |  |  |  |  |
| Ins/Ins | 48 | *18.53* | 18 | *15.93* | *0.647* | 1.20 (0.66-2.17) |
| Ins/Del | 123 | *47.49* | 57 | *50.44* | *0.680* | 0.89 (0.57-1.38) |
| Del/Del | 88 | *33.98* | 38 | *33.63* | *1.000* | 1.02 (0.64-1.62) |
| Ins | 219 | *42.28* | 93 | *41.15* | *0.842* | 1.05 (0.76-1.44) |
| Del | 299 | *57.72* | 133 | *58.85* |  |  |
| **+3003C/T** |  |  |  |  |  |  |
| CC | 2 | *0.77* | 2 | *1.77* | *0.588* | 0.43 (0.06-3.10) |
| CT | 57 | *22.01* | 21 | *18.58* | *0.543* | 1.24 (0.71-2.16) |
| TT | 200 | *77.22* | 90 | *79.65* | *0.699* | 0.87 (0.50-1.49) |
| C | 61 | *11.78* | 25 | *11.06* | *0.888* | 1.07 (0.65-1.76) |
| T | 457 | *88.22* | 201 | *88.94* |  |  |
| **+3010C/G** |  |  |  |  |  |  |
| CC | 74 | *28.57* | 31 | *27.43* | *0.920* | 1.06 (0.65-1.73) |
| CG | 127 | *49.03* | 60 | *53.10* | *0.543* | 0.85 (0.55-1.32) |
| GG | 58 | *22.39* | 22 | *19.47* | *0.624* | 1.20 (0.69-2.07) |
| C | 275 | *53.09* | 122 | *53.98* | *0.888* | 0.96 (0.71-1.32) |
| G | 243 | *46.91* | 104 | *46.02* |  |  |
| **+3027A/C** |  |  |  |  |  |  |
| AA | 1 | *0.39* | 1 | *0.89* | *1.000* | 0.43 (0.03-7.00) |
| AC | 24 | *9.27* | 15 | *13.27* | *0.330* | 0.67 (0.34-1.33) |
| CC | 234 | *90.35* | 97 | *85.84* | *0.863* | 0.88 (0.45-1.74) |
| A | 26 | *5.02* | 17 | *7.52* | *0.240* | 0.65 (0.35-1.22) |
| C | 492 | *94.98* | 209 | *92.48* |  |  |
| **+3035C/T** |  |  |  |  |  |  |
| CC | 208 | *80.31* | 88 | *77.88* | *0.689* | 1.16 (0.68-1.25) |
| CT | 47 | *18.15* | 24 | *21.24* | *0.578* | 0.82 (0.47-1.42) |
| TT | 4 | *1.54* | 1 | *0.88* | *1.000* | 1.76 (0.19-15.90) |
| C | 463 | *89.38* | 200 | *88.50* | *0.823* | 1.09 (0.67-1.80) |
| T | 55 | *10.62* | 26 | *11.50* |  |  |
| **+3142C/G** |  |  |  |  |  |  |
| CC | 57 | *22.01* | 22 | *19.47* | *0.680* | 1.17 (0.67-2.02) |
| CG | 128 | *49.42* | 60 | *53.10* | *0.590* | 0.86 (0.55-1.34) |
| GG | 74 | *28.57* | 31 | *27.43* | *0.920* | 1.06 (0.65-1.73) |
| C | 242 | *46.72* | 104 | *46.02* | *0.920* | 1.03 (0.75-1.41) |
| G | 276 | *53.28* | 122 | *53.98* |  |  |
| **+3187A/G** |  |  |  |  |  |  |
| AA | 122 | *47.10* | 53 | *46.90* | *0.920* | 1.01 (0.65-1.57) |
| AG | 112 | *43.24* | 48 | *42.48* | *1.000* | 1.03 (0.66-1.61) |
| GG | 25 | *9.65* | 12 | *10.62* | *0.920* | 0.90 (0.44-1.86) |
| A | 356 | *68.73* | 154 | *68.14* | *0.920* | 1.03 (0.73-1.44) |
| G | 162 | *31.27* | 72 | *31.86* |  |  |
| **+3196C/G** |  |  |  |  |  |  |
| CC | 122 | *47.10* | 57 | *50.44* | *0.632* | 0.87 (0.56-1.36) |
| CG | 110 | *42.47* | 46 | *40.71* | *0.842* | 1.08 (0.67-1.68) |
| GG | 27 | *10.42* | 10 | *8.85* | *0.777* | 1.20 (0.56-2.57) |
| C | 354 | *68.34* | 160 | *70.80* | *0.560* | 0.89 (0.63-1.25) |
| G | 164 | *31.66* | 66 | *29.20* |  |  |
